# A Qualitative Approach to Help Adjust the Design of Management Subjects in ICT Engineering Undergraduate Programs through User Experience in a Smart Classroom Context

**DOI:** 10.3390/s21144762

**Published:** 2021-07-12

**Authors:** Josep Petchamé, Ignasi Iriondo, Eva Villegas, David Fonseca, Susana Romero Yesa, Marian Aláez

**Affiliations:** 1 Department of Engineering, Universitat Ramon Llull (URL), La Salle, 08022 Barcelona, Spain; ignasi.iriondo@salle.url.edu (I.I.); eva.villegas@salle.url.edu (E.V.); 2 Department of Architecture, Universitat Ramon Llull (URL), La Salle, 08022 Barcelona, Spain; david.fonseca@salle.url.edu; 3 Faculty of Engineering, Universidad de Deusto, 48007 Bilbao, Spain; sromeroyesa@deusto.es; 4 Faculty of Law, Universidad de Deusto, 48007 Bilbao, Spain; marian.alaez@deusto.es

**Keywords:** COVID-19, emergency remote teaching, engineering education, first day of class, higher education, ICT, information systems, smart classroom, user experience

## Abstract

Qualitative research activities, including first-day of class surveys and user experience interviews on completion of a subject were carried out to obtain students’ feedback in order to improve the design of the subject ‘Information Systems’ as a part of a general initiative to enhance ICT (Information and Communication Technologies) engineering programs. Due to the COVID-19 (corona virus disease 2019) pandemic, La Salle URL adopted an Emergency Remote Teaching tactical solution in the second semester of the 2019–2020 academic year, just before implementing a strategic learning approach based on a new Smart Classroom (SC) system deployed in the campus facilities. The latter solution was developed to ensure that both on-campus and off-campus students could effectively follow the course syllabus through the use of new technological devices introduced in classrooms and laboratories, reducing the inherent difficulties of online learning. The results of our findings show: (1) No major concerns about the subject were identified by students; (2) Interaction and class dynamics were the main issues identified by students, while saving time on commuting when learning from home and access to recorded class sessions were the aspects that students considered the most advantageous about the SC.

## 1. Introduction

Knowledge in a specific field, the state of the art of technology, as well as learning techniques evolve year after year, so continuous adjustments to undergraduate programs are required. In 2020 the COVID-19 (corona virus disease 2019) pandemic added another piece to the puzzle, a threat that would become an opportunity to transform education [1], which involved a switch from face-to-face (F2F) to remote teaching [2], students’ performance [3] or the redefinition of the role of the instructors [4,5]. When focusing on ICT (Information and Communication Technologies) engineering programs, as in most engineering programs, a multidisciplinary approach is recommended [6,7]. In this research we propose the incorporation of undergraduates’ user experience to help refine subjects based on their perceptions about the salient items that they observed on completion of their learning experience. A specific element to analyze was the experience that students had had once used the Smart Classroom (SC) technology in the context of the subject. SC consists of a technological solution adopted by La Salle URL (Universitat Ramon Llull) to cope with the COVID-19 pandemic [8] that according to a previous research study was positively valued by students [9]. The subject ‘Information Systems’, taught exclusively on the ICT Management Engineering program, one of the seven ICT engineering programs of the Institution, was chosen as a pilot experience subject for this work since it combines technological and management topics.

The final goal of this work was to enhance the pre-existing teaching model of business subjects in ICT engineering programs through the incorporation of students’ feedback. Firstly, a description of the whole model is provided, then the techniques used to collect information are presented from a dual-fold perspective. Secondly, the evidence obtained from students’ perceptions is analyzed to combine their feedback with the other mechanisms previously implemented in the continuous redesign and adjustment of the subject ‘Information Systems’. Thirdly, information about students’ experience when using a SC was collected in order to adjust and improve its usage in future academic years. Data was collected by means of a Socratic technique and open-ended questions, resulting in a qualitative approach to evaluate both the subject and the use of the SC. Fourth-year ICT engineering students are highly skilled in technologies, so issues and concerns specifically linked to the use of a new technological approach such SC should not appear, as confirmed in the results of this research.

The novelty of this research was the incorporation of engineering students’ opinions to refine the design of management subjects through two feedback activities, one on the first day of class and another on completion of the subject to collect their salient opinions on the subject. Although some previous studies had focused on collecting information about student’s opinions of SC by a quantitative approach [10,11], we chose a qualitative approach that allowed us to collect more spontaneous feedback responses.

## 2. Research Background

Most ICT engineering undergraduate programs include topics that, according to the initial perception of students, may appear quite remote from their areas of interest. However, cross-competencies and business and management knowledge are increasingly sought-after in the labor market [12] so specific knowledge in engineering and management is a fundamental part of many engineering programs. Consequently, topics related to business, basic management, soft skills, and other generic competences are included in some subjects, or even conceptualized as a subject itself. Along this line, a similar approach is applied in most disciplines and contexts [13,14]. Therefore, we believe that more effort is needed to make management subjects as interesting and engaging as possible for engineering students in order to increase their interest in those topics.

The majority of recent undergraduates are ‘digital natives’, according to the terminology coined by Prensky [15]. Today, undergraduate students live in a world where the use of the Internet and other ICTs is widespread, despite the fact that not all of them have the same level of competence in this area [16,17,18]. Then, it is likely that this generation can learn in a different way, compared with their predecessors [19,20]. However, generations born before the ‘digital natives’ can become highly skilled in ICT by means of the proper training and experience [21]. Consequently, neither are all ‘digital natives’ highly skilled in ICT, nor are all those from older generations lacking in ICT knowledge. Therefore, in all cases, people that are experts in ICT can take full advantage of the potential that these technologies provide, e.g., [22]. In the specific field of education, digital technology can leverage student experience in different facets [23], making digital technology a key element in higher education [24].

### 2.1. Context of the Study

According to this reality, in the 2018–2019 academic year La Salle URL adopted the ‘New Learning Context’ (NLC) to enhance student learning [25,26]. The main reasons for implementing this new approach were: (1) most new entrants as undergraduates are used to working with technology tools and in digital environments; (2) the available ICT technologies can improve different aspects of learning; (3) the modern approaches of learning and teaching help acquire skills and knowledge more effectively. For this purpose, the NLC establishes a pedagogical framework to achieve specific outcomes by means of active learning through different learning areas (welcoming, seminar, workshop, project, and closure). Although the NLC is out of the scope in this paper, it is a key point to understanding why La Salle URL engineering programs are undergoing an extensive redesign process. In this context the deployment of a replicable design methodology for different subjects would be interesting.

The research presented in this paper was carried out in the context of a fourth-year subject that includes engineering and management topics, with the aim of developing a model to be replicated in different third- and fourth-year management subjects. All the previously mentioned ideas were considered in the redesign of the subject ‘Information Systems’. A Project Based Learning (PBL) methodology was adopted [27], following the constructivist logic while working in a collaborative learning context. Besides, traditional classes were transformed into short sessions in the form of a ‘pill classes’ specifically designed to promote students’ participation, as explained in [28]. What is more, this redesign was implemented to reinforce students’ active learning [29,30], along the line of the NLC. It should be highlighted that a learning environment which applies PBL enables students to work a similar way as many engineers do in most of the cases when they enter the labor market [31,32]. On the other hand, competences such as teamwork, written and verbal communication skills, critical thinking, and so on, are highly valued in the labor market, resulting in increasing students’ employability according different studies, e.g., [12]. So, different tasks and activities where included in the new design of the subject to teach and develop these competences, such as the introduction of Self- and Peer to Peer assessment activities [33].

### 2.2. Technologies Deployed at La Salle URL to Cope with the COVID-19

In 2020 the nature of the COVID-19 pandemic seriously affected many aspects of people’s lives, particularly with regard to social interactions and public health [34], besides putting at risk most worldwide economies [35]. As a consequence of the quick and easy propagation of the virus, governments took measures to minimize its effects on health (limiting and restricting mobility, isolating ill people,…) [36,37,38]. Consequently, in the specific area of education measures were introduced to prevent the virus from spreading [39]. However, the effects of the disease on people, besides other consequences derived from some of restrictions adopted, have had a harmful effect on people’s health, provoking feeling of anxiety and stress [40,41,42]. Focusing on education, most educational institutions restricted and limited the physical access to their educational facilities because of governmental decisions adopted to constrain most of the activities that involved direct social interactions of any type. As a result, online learning was thought to be a viable solution to cope with the pandemic constraints [43,44]. The immediate reaction of educational institutions was to implement emergency remote teaching (ERT) solutions. These new formats consisted of temporarily switching from F2F class sessions to remote teaching [45], which became a challenge for most of the agents involved in the change [46,47,48]. The competences and attitudes of the teaching staff were two key points when they were forced to change from F2F to ERT, specifically: (1) their competences in ICT, including digital literacy and teaching skills in an online environment; (2) their attitudes, such as perceived threat, their ability to develop new skills or a positive approach to challenge [5]. Regarding the students, they switched to remote learning which allowed them to maintain their educational training despite mobility restrictions. However, different challenges arose when attending classes off campus, such as distractions or less effective student-instructor and student-student interactions [9]. In addition, different research works have reported issues in other areas, such as their wellbeing [49], learning capabilities or engagement levels [50].

The pedagogical system of La Salle URL evolved from an ERT solution implemented at the beginning of the second semester of 2019–2020 academic year to a SC deployment before the start of the 2020–2021 academic year [9]. In other words, it implied moving from a tactical solution (ERT) to cope with the COVID-19 pandemic, to a strategical approach based on the SC format. This latter strategy consisted of a large investment in technology with the aim of leveraging students’ experience in the case that the undergraduates had to stay at home. To do that, classrooms and laboratories were equipped with different hardware devices, such as a sound system, an image system and a ‘Smart-Board’. In addition, a software solution to allow a proper interaction with different electronic devices completed the solution. The SC format was introduced to enable off-campus students to follow classes in real time to make the learning experience as similar as possible to attending classes physically in the classrooms and laboratories at the campus facilities. In fact, the ERT solution was effectively implemented thanks to two elements: (1) the existing infrastructure in the campus facilities to provide services to a few existing online programs, infrastructures that were simply adapted and scaled to meet the new requirements; (2) the instructors and the engineering students were able to easily shift to remote teaching and learning because of their background and abilities to adapt quickly to the new tools. It should be said that although students had been taught on a F2F basis until the outbreak, all of them were already familiar with a Learning Management System (LMS). In the preexisting context (F2F), LMS was used to consult the syllabuses, contents and class materials of subjects, grades, uploading homework or just accessing to information of their interest.

**Figure 1:**
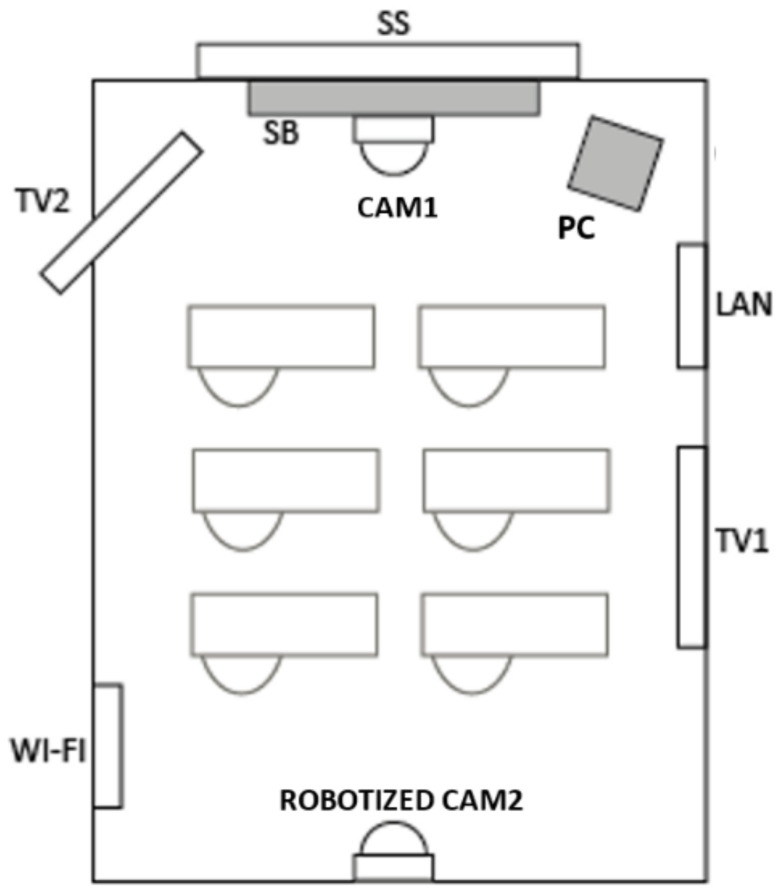
Technological devices deployed in a standard ‘Smart Classroom’: Sound system (SS); Smart-Board (SB); Camera 1 (CAM1); Robotized Camera 2 (CAM2); Television 1 (TV1); Television 2 (TV2); Local Area Network (LAN); Wi-Fi; Personal Computer (PC).

The 2020–2021 academic year constituted a second stage, since strategically La Salle-URL decided to focus on a new approach which was much more consistent with the initial F2F modality even considering the COVID-19 pandemic threats [9]. Hence, a new model based on SC as shown schematically in Figure 1 was implemented which aimed to maintain the advantages of F2F learning [51]. The SC format was thought to be resilient enough to cope with commuting restrictions that could affect students and instructors, characterized by incorporating hardware devices inside more than sixty classrooms and laboratories [52], allowing different possibilities as explained in a case study [8]. SC is a concept characterized by the introduction of electronic devices at classrooms, such as cameras, televisions, smart-boards, or sensors [53,54,55,56]. SC has a huge potential due to its technological nature. Then, it can be said that SC is an instrumental tool to allow smart education [55,57], that may allow personalized learning and the possibility of learning anytime and anywhere [55]. Besides, SC can be a very convenient instrument when using project or problem-based learning techniques [58]. As pointed out in [53,54,59], SC makes possible a blended model mixing F2F on-campus and off-campus classes.

**Figure 2:**
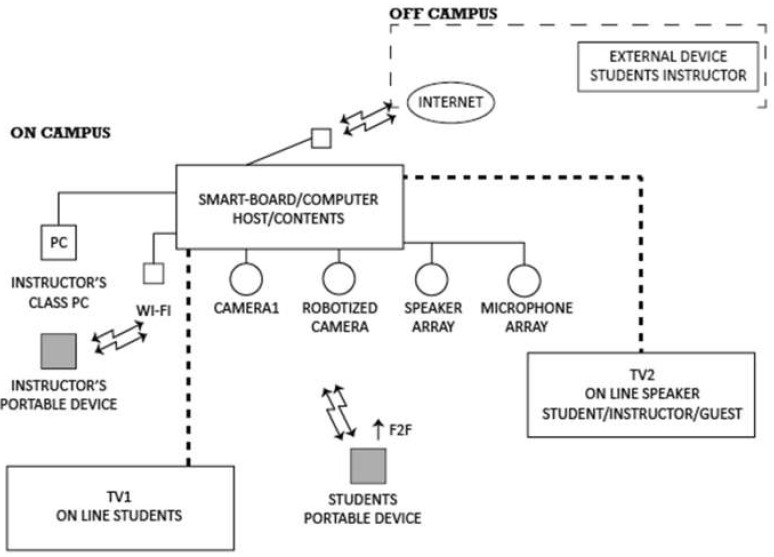
User interaction with the technology deployed in a ‘Smart Classroom’.

From the user perspective of the student and as shown in Figure 2, the system can be described as compounded by different parts, as follows:

- Sound system: Microphones and speakers. Both devices allow the sound interaction between people that are physically in the classroom (on-campus) and those at home (off-campus). To have a complete interaction, those at home must use a device that includes their own sound system.
- Image system: Two cameras (one of the cameras is a robotized camera that can follow the instructor while moving in the classroom class), plus other camera that shows a full view of the classroom. Through the Smart-Board, the instructor decides which view is shown to students attending classes off campus. Two TV sets complete the hardware devices to provide images. Once again, to allow complete interaction, people at home must use a device with their own audiovisual system.
- Smart-Board: In fact, it is a computer with a huge screen that acts as a blackboard (or whiteboard) or as a projector. The screen is a touchable board that allows drawing on its screen, by means of just writing directly on the screen or by writing from the different devices connected to the class session (under permission of the instructor).
- Software to allow a virtual connection between the classroom Smart-Board and all the other systems located on- or off-campus.

**Figure 3:**
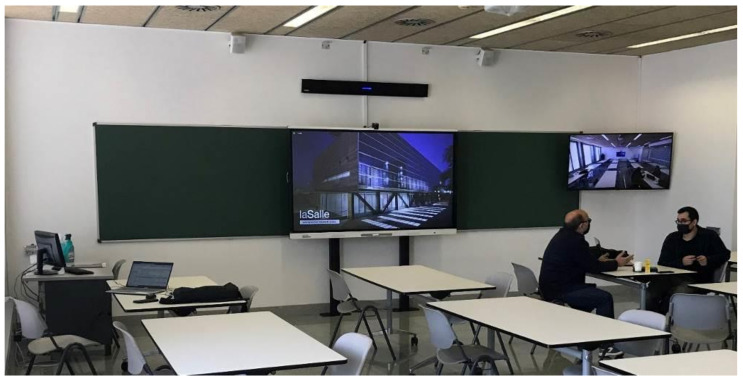
‘Smart Classroom’: ‘Smart-Board’, Sound system, Camera1 and TV1.

**Figure 4:**
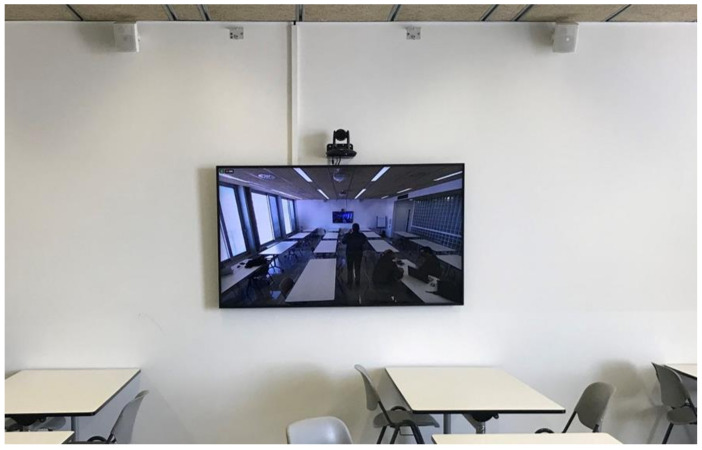
‘Smart Classroom’: Sound system, Robotized Camera2 and TV2.

In addition, some supplementary changes were made to allow remote access to several devices located physically in the campus facilities, such as laboratories. This latter casuistic is not described in this paper, since ‘Information Systems’ does not require access to any additional devices, except the ones already described. Finally, to complete a whole picture of how looks a typical ‘Smart Classroom’ at La Salle URL, two actual photographs are shown in Figure 3 and Figure 4.

It should be highlighted that the initial goal of the SC format was to offer an on-campus experience despite being off-campus thanks to an online solution which could effectively overcome the main issues that traditional online formats have. In other words, the aim was to make the off-campus experience as similar as possible to the on-campus experience. To accomplish this objective, some features of the specific SC deployment are briefly explained in the next lines. The robotized camera tracks the instructor, trying to give a feeling of a greater interaction with the instructor with students who are at home. The other camera is thought to offer a general vision of the classroom to off-campus students. One of the TV devices shows all the off-campus students, thought to integrate off-campus and on-campus students in the class sessions. The other TV device shows the off-campus student that is interacting live with all the other people following the class session. The ‘Smart-Board’ enables instructors to show presentations, videos, or files on its screen. Besides, it can act as a white or black board. On a regular basis, the instructor shows and writes contents on the screen of the board. Students may show or write content on the board under instructor permission. Additionally, the system allows recording class sessions. As a result of the policy of the Institution, recorded classes are available to all students for a limited period. This option enables students to review the class content in case they wish to go over anything when studying contents. Hence, all this technology enables that students to attend classes off-campus. Besides, this technology has proved to be useful when the instructor has been unable to physically come to campus as it has enabled them to teach class sessions from their own home if needed [8].

### 2.3. Teaching ‘Information Systems’ at La Salle URL in the Context of ‘Smart Classrooms’

In the 2019–2020 academic year, a fall-semester fourth-year ICT engineering subject named ‘Information Systems’ was redesigned. This subject, as all the subjects given in the seven official ICT engineering programs, is taught on-campus in a F2F modality. In fact, most programs at La Salle URL are officially recognized as F2F instruction modalities. In the very first academic year of the new design, the subject was taught on a F2F format with all students attending classes physically in the classrooms.

In September 2020 classes started in a context characterized as commuting restrictions were lifted due to the positive evolution of the pandemic. This situation affected students, instructors, and other people that worked in the Institution. To cope with this situation the subject shifted to a new teaching and learning format based on SC, since this new strategy had been adopted at La Salle URL.

Classes were conducted by two highly skilled instructors, both working in management positions in the Information Systems area, besides teaching part-time classes at a master’s degree level. Instructors taught classes on-campus throughout the fall semester. From the very first day students had two options available: attending on-campus classes, inside the classroom; or attending online off-campus classes once students were remotely connected to the class sessions. The subject was organized in 14 weekly sessions of 150 min, including a final project presentation. Classes were split into two parts: the first one, with short explanations given by the instructor, designed to stimulate students’ participation by trying to encourage questions from students, or through questions asked directly by instructors to the students; and a second part designed to work under the PBL logic. Then, during the second part of each class session students worked in groups of three or four, while the instructors acted as consultants [28]. The PBL activity started from the analysis of a real case in which the deployment of an information system initially failed. Next, the students had to discover the reason why it had failed and then propose a feasible solution. This task was developed over twelve sessions. In session 10, all groups gave a mock presentation to their classmates and to one of the instructors with their preliminary findings in order to obtain feedback and refine their final conclusions. Finally, each group presented their final results and conclusions in the last class session, giving an oral presentation in front of all the students and a judging panel that included three instructors. The instructors assessed the students’ individual performance in the presentation as well as in the question and answer session. In addition, the final grade included a self-evaluation and a peer-to-peer weighting [33]. In the case that students were attending off-campus classes, a software application enabled them to join the class in real time [8]. This platform also enabled the creation of virtual groups where students and instructors could interact.

SC allow leveraging teaching and learning class activities [53,56,58,60]. However, since this technological solution was deployed just a few days before starting the 2020–2021 academic year, not all the potential features derived from the SC were exploited to take advantage of the new possibilities offered by the new format. The approach of the subject in this new context, in line with the objective of La Salle-URL in the 2020–2021 academic year, was simply to make the learning experience for off-campus students as similar as possible as that of the on-campus students.

### 2.4. Integrating Students’ Feedback in a Model to Adjust the Design of a Subject

**Figure 5:**
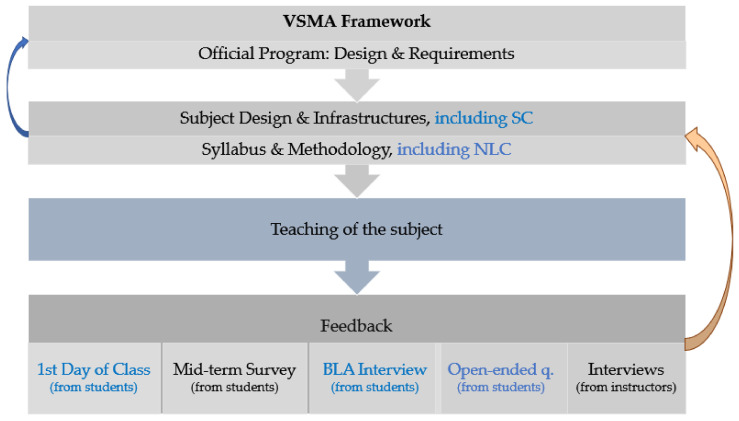
Continuous adjustment of the subject once feedback from the students is collected.

This study was focused on enhancing the design of a model based on the continuous adjustment of a subject once each cohort of students had completed the subject, as shown in Figure 5. This diagram includes different elements: the pre-existing elements are colored in black, while the new ones presented in this research work are colored in blue. In short, the different stages of the proposed model are: (1) To seek from the National Accreditation Board validation of a new official program that includes all the curriculum design, according to its framework for the validation (ex-ante accreditation), monitoring, modification and ex-post accreditation of recognized degree programs (known by its Spanish acronym VSMA) [61]; (2) If the proposal is accepted by the National Accreditation Board, the new program is implemented through different subjects according to the different methodologies, deploying new infrastructures in the campus facilities, if required; (3) To teach the different subjects of the program; (4) To receive and analyze feedback from instructors and students to allow the fine-tuning of each one of the subjects, while keeping the mandatory core elements officially approved. Hence, a user experience assessment on 2019–2020 and 2020–2021 students was carried out to adjust and refine the design of the subject once the collected data had been processed. This model was implemented with the aim of being potentially replicable in other third- and fourth-year management subjects imparted in the seven ICT engineering programs at La Salle URL. This continuous adjustment was initially planned to be carried out on an annual basis once students had completed all their class activities, including assessments. In order to complete the whole picture, if the changes to be made in the subject as a result of the analysis of possible improvements (brown arrow in Figure 5) are not consistent with the official degree program, these changes should go through a process of modification (M) or monitoring (S), according to the VSMA framework (blue arrow in Figure 5).

In addition, SC was introduced into the model since it became a new way of teaching and learning in the Institution, providing new interesting possibilities from a didactic point of view.

Different techniques collect students’ feedback, through either open-ended or closed-ended questionnaires [62]. The first day of class is a crucial day that may have an impact on several elements, such as building students’ expectations [63], attitudes [64], engagement [65] or motivation [66,67]. In fact, most teaching books have a chapter about the first day of class [68,69]. Some surveys have been performed by researchers to detect students’ preferred activities on the first day of class [70,71,72,73,74]. When instructors identify what the students’ preferred activities are, instructors can incorporate them into the subject, once aligned with the goals of the subject.

On the other hand, students’ feedback can be collected on completion of a subject. User Experience (UX) is a topic that has been defined by highlighting different elements and perspectives, as reflected by Laws et al. [75]. According to ISO (the International Organization for Standardization), user experience can be defined as the ‘person’s perceptions and responses resulting from the use and/or anticipated use of a product, system or service’ [76]. As explained in Tullis and Albert [77], some options must be chosen depending on the research, as done: in terms of study goals, formative experience; in terms of user goals, satisfaction; and in terms of choosing the right metrics, self-reported metrics. Then, assessing user experience with a proper technique may be a suitable way to obtain students’ feedback about classes. Laddering Theory has to deal with one-to-one in-depth interviews to obtain users’ evaluations of products or services, in order to identify the most important attributes [78,79]. Bipolar Laddering (BLA) is a tool designed to extract value from user experience by means of Socratic survey, once the user has completed the experience related with a product or a service [80]. BLA can be described as a process that requires different steps [80,81], as follows: Firstly, using a Socrates’ Tabula Rasa (i.e., without previous conditionings), users spontaneously identify the strong and weak points of the experience according to their perceptions and they justify their selection; secondly, users assess each one of the identified elements (out of 10; being 0 no satisfaction, extending to 10, the maximum level of satisfaction); finally, users are asked to give their suggestions about how to improve each one of all the items that they have mentioned.

## 3. Methods

As formerly mentioned, the aim of this research was to obtain data to adjust the redesign of a subject while analyzing issues derived from the implementation of SC. Our final research objectives were: (1) To obtain information from the students’ viewpoint about the redesign of a subject started in 2019–2020, ‘Information Systems’; (2) To propose a replicable methodology, as shown in Figure 5, to readjust subjects including a user experience approach to obtain qualitative feedback from students; (3) To detect positive and negative effects derived from the use of a SC format from the undergraduates’ viewpoint.

To perform this qualitative research, a user experience approach was chosen to get students’ feedback. Two techniques, Bipolar Laddering (BLA) and Emotional Appraisal have been used in this research due to the linkages that exist between them [82,83]. BLA interviews, a specific question about utility and two questions about SC are based on open-ended formats which minimize possible biases [62]. On the other hand, data about preferred activities to be done the very first day of class could provide very valuable information about eventual students’ concerns related to SC, besides adjusting the final design of the subject. Finally, two specific questions about SC were included in this research to provide feedback about this topic. Hence, some different instruments were used to capture data needed to analyze users’ perceptions about the subject and the SC.

### 3.1. Participants and Procedure

This research includes undergraduate students from two different cohorts enrolled in the subject ‘Information Systems’ in two consecutive academic years, thirteen in 2019–2020 and eighteen in 2020–2021 academic year. The only difference that existed between both cohorts was the implementation of SC, as previously explained. Additional details about participants that responded the questionnaires and interviews are shown when presenting results, as not all the research techniques were carried out in both cohorts. The second cohort experienced the on-campus and off-campus SC. In order to obtain feedback about the subject by means of a user experience appraisal, the student must have finished the whole experience. So, the initial schedule was to perform the students’ interviews in February 2020. However, since the pandemic was declared at that moment, we preferred to postpone the surveys to avoid potential biases derived from the critical situation (see Section 3.3).

A User Profile Test to check potential differences in each one of the two cohorts to establish criteria to select a proper sample for each specific cohort was not required to be done, since all students of ‘Information Systems’ can be considered homogeneous: (1) All the 2019–2020 students attended class in the classroom at the campus physically, while all the 2020–2021 undergraduates experienced a SC class format, attending some of them on-campus and off-campus classes due to some commuting restrictions during that semester; (2) all undergraduates were studying an ICT engineering degree. Therefore, asking students to voluntarily complete a questionnaire implied no issues in terms homogenously concerns about the resulting sample, once reached a minimum number of respondents.

This qualitative research has been planned according the guidelines and criteria presented in previous studies [84,85,86,87,88,89]. So, as presented in [84], having selected the aim of the research, the next steps were to identify potential respondents, decide the methods to collect data, and finally select the analysis methodology. Techniques to measure user experience have been deployed in other studies to collect students’ experiences in an educational context, e.g., [90,91,92]. The analyzed data was directly collected from students by means of questionnaires and interviews. The context from where data were extracted has been clearly explained, students of a fourth-year ICT engineering undergraduate program while attending the subject ‘Information Systems’, being the sample of surveyed students homogeneous when compared with the total number of enrolled students. Once data has been collected, the items have been analyzed separately by three different researchers who finally came to a consensus, a way of triangulation to increase the validity of the results [84]. When presenting results in this research, all students’ opinions were transcribed literally.

### 3.2. An Empirical Study: First Day of Class, What ‘Likes’ and ‘Dislikes’

An empirical study was performed the first day of class with all the 2020–2021 students that were on-campus at the classroom. Once the very first session of class started, the subject coordinator, who was already known by all the students, welcomed all the students briefly. Next, he introduced the instructor that was going to give the initial class, just mentioning his name, and announced that an introductory activity was going to be performed. After that, an anonymous survey was handed out to all the students that were in the classroom, asking them to complete the form on a voluntary basis. The survey included data about ‘age’ and ‘gender’, besides two open questions, each one to be answered on one side of the paper. In the first side of the sheet of paper the open-ended question was ‘What things would you like an instructor to do on the first day of class of this subject?’, while in the other side of the paper the question was ‘What things that an instructor does on the first day of class of this subject do you dislike?’ Students were requested to answer the questions with a short sentence, if possible, just writing a few numbers of words (five or less). Once researchers collected the forms, answers were reworded to uniformize students’ responses. Three researchers did the job on an individual basis. The next step consisted of reaching agreement on the results, once compared, and discussed. Thanks to the latter task, items were analyzed and finally aggregated to show percentages of occurrence.

### 3.3. User Experience

Different techniques have been used to collect students’ user experience. In this research we have gathered data from a user experience viewpoint from two different students’ cohorts: 2019–2020 and 2020–2021. All the interviewed students signed a form allowing the use of the collected data for research purposes, besides authorizing the recording of their opinions of the BLA interview, the open-ended questions, and the Emotional Appraisal. A total number of ten respondents gave their opinion about experiencing ‘Information Systems’, five of each cohort, albeit 32.3% of the total enrolled students. The number of people to be interviewed to get accurate results is five according to Nielsen [93]. However, this number should be increased at times depending on the degree of homogeneity of the universe and representative issues of the chosen sample [94]. It should be noted that in this research the surveyed sample is homogenous with the whole universe of students. The BLA technique used in this research is based on interviewing respondents in an open-ended basis, without limiting the number of answers of the interviewed people. This latter design decision has a great impact on the findings, since even with relative small samples, such as ten samples most of the salient items can be obtained, as posited in [95]. As found in [96] this latter finding is consistent with previous research works [97,98,99,100].

- Collecting results from the 2019–2020 cohort was done in December 2020 to five of the 13 enrolled students (38.5%). The mean age of the sample was 21.06 years with a variance of 0.64, three males (60%) and two females (40%). Students received two e-mails asking for their collaboration to do a survey about the Information Systems subject. All the interview activities were performed by a very skilled instructor in the use of the UX techniques who had not had any kind of relationship with the subject, to avoid any possible biases.
- Collecting results from the 2020–2021 cohort was performed in January 2021 to five of the eighteen enrolled students (27.8%). In terms of age, the mean of the sample was 22.2 years with a variance of 5.76, all men. In the last class session, the teacher told the students that they would receive an email once their grades had been published and they were invited to a voluntary meeting to give their opinion on the subject. The interviewer was the same instructor that surveyed the first cohort.

#### 3.3.1. Bipolar Laddering

During each semester, all engineering undergraduates at La Salle URL are asked to give their opinion about all the subjects in which are enrolled by means of what is named a ‘Satisfaction Questionnaire’. This survey is completed online, and students rank different statements. Besides, the questionnaire includes a blank space at the bottom of the form where students can add observations about whatever they want. The results obtained are useful ‘to raise a flag’ in case that some non-detected issues are affecting students. Most of comments deal with additional information about some of the previously ranked statements. So, this technique can be useful to detect some general issues, while providing results by means of statistical tools.

In contrast, data obtained by means of the BLA technique cannot be treated statistically, since respondents give their own opinion about a specific item, not necessary a coincident one. Nevertheless, possible biases derived from the redaction of the question are minimized, and genuine perceptions about a particular topic of interest can be collected. Hence, BLA can be a very useful technique to obtain students’ perceptions of a subject from their user experience as learners and then use this information to improve it.

Methodologically, in order to show findings and once all the students’ ideas had been analyzed, a rewording process is carried out to write a clear statement linked to each idea. This task was done by three of the authors of this research, who wrote the final statements. Once done that, each one of the statements is grouped according one of the next criteria, as follows: (1) Positive common elements, or positive items mentioned by at least two users; (2) Positive particular elements, or positive items mentioned by just one user; (3) Negative common elements, or negative items mentioned by at least two users; (4) Negative particular elements, or negative items mentioned by just one user. This technique has been used to assess different user experiences in diverse fields e.g., [92,101], including teaching experiences [90,102,103,104].

#### 3.3.2. Open-Ended Questions

Open-ended questions offer the advantage of getting spontaneous answers from the surveyed people, besides minimizing the bias resulting from the closed-ended questions [62]. Hence, to research on three specific issues of interest, three open-ended questions were asked to the students: a first one about the perceived usefulness of the subject to all the students and two additional questions about the SC format that were only asked to the respondents of the 2020–2021 cohort, since they were the only ones that had experienced the SC format. The open-ended questions were as follows:

- Question 1. Do you think that ‘Information System’ has been a useful subject in the context of the ICT Management Engineering program?
- Question 2. What is your opinion about the ‘Smart Classroom’ system deployed at La Salle URL to cope with the COVID?
- Question 3. Once you have experienced the ‘Smart Classroom’ classes… Do you think that you learn the same by experiencing the classes on- and off-campus?

#### 3.3.3. Students’ Emotional Appraisal

Emotions may have a great effect on human beings, as posited in different works [105,106]. When focusing on educational science different studies have analyzed the impact of emotions on students’ motivation and interest or academic achievement [107,108], since emotions play an important role in learning [109]. Henceforth, getting data about students’ emotions once they have experienced the whole learning process of a specific subject may provide relevant information to the instructors to adjust the teaching process in the next future.

Appraising emotions once a product or service has been experienced by the user may provide very valuable feedback. Schmidt-Atzert [110], implemented a questionnaire based on different opposite pairs of feelings to be assessed by the user. The appraisal process consists in selecting one of the five points-scale that extents between both pairs of emotional states (100%, associated to the positive feeling; 75%; 50%; 25% and 0%, associated to the negative feeling), which are shown in Section 4.4. Once the user finishes this process, the outcome offers a whole picture in which different pairs of related positive and negative emotions are showed [91,110,111].

## 4. Findings and Results

Once the different goals to achieve in this research have been previously defined, the results are presented in this section. Four different instruments were used to obtain information related with this research. All students that physically attended class were asked to complete voluntarily an anonymous survey about the preferred and the undesired activities to be done on the first day of class. To complete the other three instruments, an e-mail was sent to all students on the completion of their experience, inviting them to participate in a survey. Once five positive answers of each cohort were received, the interviews were done.

### 4.1. An Empirical Study: First Day of Class, What ‘Likes’ and ‘Dislikes’

The total number of respondents that completed the survey were 13 out of 18 enrolled students in the subject in the academic year 2020–2021. The mean age of the respondents was 23.08 years with a variance of 3.90, being 69% males and 31% females.

Results were captured from a survey done by means of two open-ended questions and are shown in Table 1. Students answered on what they liked and what they disliked, in terms of activities and tasks to be done the first day of class of the subject ‘Information Systems’.

Findings are consistent with previous empirical findings such as [70,73,74] in terms of the items (activities) ranked on the top positions of ‘likes’ and ‘dislikes’. However, percentages vary, and some elements such as ‘Motivating students’ and stressing the ‘Utility and objectives of the subject’ can be highlighted in our results. On the other hand, no activities or issues related with SC or about the COVID-19 pandemic were reflected in the students’ answers.

### 4.2. Bipolar Laddering (BLA)

A BLA interview was done to both students’ cohorts, 2019–2020 and 2020–2021. Results are shown in terms of positive and negative perceptions. Besides, both data sets are grouped depending on whether they are mentioned by two or more students or just being cited by a single student.

- BLA: 2019–2020 academic year results

Table 2 shows four positive elements cited by two students or more. The table includes a succinct description of each statement and its assessing out of 10. It also includes the average score of each item, and the percentage of students that have mentioned the statement, under the label ‘mention index’. Items have been ranked and presented in the table according to their average score. The only statement that has been mentioned by all the students (‘Instructors work in the Information System field’) achieved the maximum average score (9.60). All items have a very low variance (0.24 or less), except the item about PBL (variance 1.25).

Different suggestions were made to improve each one of the four elements. Item 19PCE1 (both instructors working in the Information System field) was considered “quite difficult to improve” in a greater extent consistently with students’ assessment. Item 19PCE2 (the subject is real approach to the labor market) could be improved by means of “including more insights, specifically linked to topics related with the market entrance”, or as mentioned by two students by “working on real cases”. Item 19PCE3 (using PBL) could be improved “if each group works in different cases” as said by two students, by “developing a real project” or by giving “less theory and more practice”. Finally, item 19PCE4 (teamwork) could be enhanced according students by “establishing a competition between groups” or by “mixing students, to increase students’ interaction”.

Table 3 includes the eight positive elements mentioned only by one of the respondents. The elements identified by the students in this category were ranked with values from 7 to 10.

Table 4 contains the only two negative elements that have been mentioned by at least two students. Despite been considered negative, both items have been scored with values quite close to 5 (in fact, 4 and 5 in each one of the elements).

Students’ comments about how to improve the item 19NCE1 (a non-suitable timetable) were: “finishing classes earlier in the evening” or “doing different class sessions of an hour and a half each per week”. On the other hand, the element 19NCE2 (all groups working in the same project) two students coincided in their appreciation by saying that it could be improved by means of “doing different projects each group”.

Table 5 contains four negative elements, each of them mentioned by a single student. It can be highlighted that an element (19NPE1, ‘an excess of traditional classes’), despite been considered negative, was assessed with a score of 6. Item 19NPE4 was reported by a student who did a great job during the course, but failed in the final presentation, as he recognized in the BLA interview.

- BLA: 2020–2021 academic year results

Table 6 shows the positive common elements identified by the students of the 2020–2021 academic year, while Table 7 shows the positive elements mentioned only by one of them. The positive common results are quite coincident with those showed in Table 2.

Students’ comments about how to improve the item 20PCE1 (both instructors working in the Information System field) were: “it is quite difficult to improve”, “giving some class sessions with both instructors teaching at class altogether”, or “inviting more instructors to teach some specific sessions”. When thinking about how to improve the item 20PCE2 (the subject has a great content), students suggested that “it is difficult to improve” or that it could be done by “including more homework about Customer Relationship Management (CRM) and Enterprise Resource Planning (ERP), while minimizing content about e-commerce, since students already learned this topic in other subjects”. Suggestions about how to improve the item 20PCE3 (using PBL) are in line with “doing more short assessing activities”, as said by three students. Item 20PCE4 (teamwork) could be improved by “working F2F instead of online” or by “keeping a better track of each one of the students to avoid free-riding”. When talking about the element 20PCE5 (interactive classes) students propose “reducing class lecture time while increasing instructor-student interactions” or “reducing theoretical explanations”.

There was only one negative common element mentioned by the students, as shown in Table 8. Students’ comments about how to improve the item 20NCE1 (Final Project centered in CRM) were: “doing a more complex project” or “focusing on CRM and ERT even more”.

Table 9 shows the negative particular items mentioned by only one interviewed student.

### 4.3. Open-Ended Questions

Respondents of the 2019–2020 cohort only answered question 1, because they did not experience SC, while the 2020–2021’s answered all the three questions. Students’ answers to the three open-ended questions are presented, as follows:

- Question 1. Do you think that ‘Information Systems’ has been a useful subject in the context of the ICT Management Engineering program?

All the students answered in line with the idea that this subject had been very useful: “Very useful. This subject enabled me to understand applied concepts, besides comprehending some tasks that I am supposed to do”; “Very useful. Very important and interesting. In the long term, I would like to manage similar projects. It is very important that this subject is taught by real information systems professionals”; “I have learnt a lot. Theory plus a real application of concepts. One of my best subjects in my engineering studies. Oriented to the labor market. Very interesting for ICT engineering students. Indispensable”; “It is really useful. Even better if some more practical elements were included”; “I t is good to know how things are done in the labor world. An interesting subject, despite it is not indispensable”; “Very useful for an ICT Management Engineering student. I believe I shall study a master program in this line”; “Very useful. It is the most motivating subject of this semester”; “Very useful. We have learnt how different departments work in the real world. This subject should be included in all seven ICT engineering programs at La Salle”; “Very useful for ICT Management Engineering students. I have learnt a lot. Very useful when thinking about accessing to the labor market. A transversal knowledge about companies and businesses is given”; “I do not know if I shall use the content learned in this subject. However, it is a very useful and applicable subject. A very good subject. It gives a whole picture about companies. Highly experienced instructors. A very motivating subject”.

- Question 2. What is your opinion about the ‘Smart Classroom’ system deployed at La Salle URL to cope with the COVID?

Student’s answers were: “Very interesting and nicely implemented. It enables us to following classes from home. I have attended a lot of classes in a F2F format, and students that were attending online, could easily interact. Very easy format to use, F2F and online”; “I prefer the F2F format. When you are attending online classes, it is much more difficult to interact and to pay attention. Despite all the commented issues, I have learnt all the explained concepts by the instructors. F2F classes are funnier that online classes”; “Something is lost when attending classes from home. Everybody is comfortable in his own home, but everything is done through a screen. It is more difficult to connect with the other students. However, it has some positive elements: you are comfortably in their home and no commuting time is spent to go to the campus facilities. Instructors can be asked easily. I think that online learning is not an issue in this subject. In subjects where a laboratory is required, this format implies some issues”; “Interacting by means of a screen is a quite cold feeling. Being on-campus in the classroom improves students’ experience compared with online formats. Students ask and interact more when they are in the classroom. At home there are a lot of distractions”; “When thinking about different online option, ‘Smart classroom’ is the best option. However, being in the classroom is better. Some weaknesses of the online format: it is quite difficult to concentrate; you are not in a close contact with the other students, influencing personal mood and attitude. I think that watching a screen during all day is not good, and I feel that on me”.

- Question 3. Once you have experienced the ‘Smart Classroom’ classes…Do you think that you learn the same by experiencing the classes on- and off-campus?

Students answered this question highlighting different ideas: “When you are at home, sometimes you are reluctant to ask questions because in some cases you are not sure if the topic to be asked has been already explained (because of possible distractions at home). When you are attending classes in the classroom you are more engaged in the class dynamics”; “attending classes in the campus facilities allow a better adaptation from instructors to each student, since it is easier for the instructor to perceive how the student reacts to class contents. Being in the classroom allows more interaction than online format. Being in the classroom adds more value to what the instructor is explain to the students. Clearly better being in a classroom than experiencing the online format in this subject. I would have enjoyed more this subject in a fully F2F format in the classroom. When learning other subjects, perhaps there is not such a different when doing classes in a classroom or in an online format”; “I have followed all classes online. I have learnt a lot because I have had instructor’s’ notes in advance. I was able to follow all the explanations without problems in this subject”; “It is easier been concentrated in the F2F format than in the online format. When classes are performed in traditional format there is no problem; when classes are based on discussions and interactions, it is not the same. When we are all in the classroom, motivation is higher and interaction is increased”; “In the online format the learning process is worse, it is slower than in the F2F format. Some positive elements of the online format are: notes and written documents are available in advance; recorded classes are available; you do not need to be fully concentrated to write what is said by instructors or students since class sessions are recorded. However, these advantages have the effect of decreasing the concentration level of the student”.

### 4.4. Emotional Appraisal

Emotional Appraisal questionnaires were done to compare results with different students’ cohorts, 2019–2020 and 2020–2021. Table 10 lists the different pairs of antonymous feelings assessed, including the total average of all the pairs of feelings, 76.36% for the academic year 2019–2020 and 85.91% for the 2020–2021 academic year.

**Figure 6:**
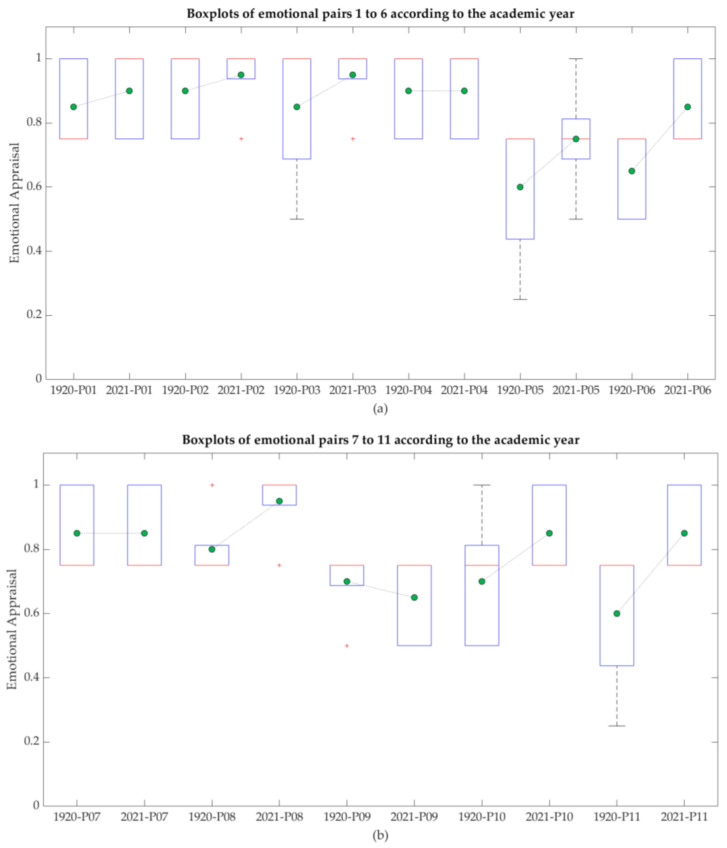
Boxplots: Comparison of 2019–2020 and 2020–2021 Emotional Pairs. The means of each group are marked with green dots. The numbering of the pairs of emotions corresponds to that shown in Table 10. The different subfigures show: (**a**) results from pairs 1 to 6; (**b**) results from pairs 7 to 11.

Figure 6 shows all the different pairs of feelings, by means of boxplots, and compares 2019–2020 and 2020–2021 results.

## 5. Discussion

This study analyzes students’ insights by means of different techniques from a two-fold perspective. On the one hand, the results obtained from the surveys and the interview activities should allow the fine adjustment of the redesign of this fall-semester fourth-year subject imparted in the context of an ICT Management Engineering program. On the other hand, gathered data should give information about students’ perceptions on the students’ learning experiences when interacting with SC with the objective of identifying positive outcomes while detecting elements that could need an adjustment. Table 11 shows a synthesis of all the items listed in Section 4, rewording and synthesizing students’ perceptions about the subject.

### 5.1. Fine-Tuning of the Subject

The first day of class survey gave information consistent with previous academic works, e.g., [70,73,74]. Students’ priorities were concerned with receiving information about the syllabus, class content, the utility of the subject and information about assessment, while beginning subject content. Getting to know their classmates was not highly ranked in terms of frequency and could be explained since they were fourth-year students, and most of them were of the same cohort and knew each other from a long time ago. Just one student proposed not to exhaust the class time, a result that in other empirical surveys is higher [70,73,74].

Results from BLA are quite consistent when comparing data from 2019–2020 and 2020–2021 academic years (see Table 11). Students greatly appreciate the subject’s content, the assessment system and the instructors’ performance. PBL is highly valued as a way of working, in line with [30,31,32]. Besides, an element closely related with the PBL logic, such as teamwork, is also mentioned as a positive element. Timetable appears as one of the weak points of the subject, mainly for students that attended on-campus classes in the 2019–2020 academic year, since classes finished quite late in the evening and they had not the option of attending off-campus classes, as 2020–2021 students had. This element is quite difficult to cope with and it is the price to pay in case of selecting instructors that work in the industrial environment as their main activity, in fact one of the most assessed items by undergraduates. The element that is considered an issue by students is the fact that all students are working on the same project. In fact, the initial approach was that working on the same project could provide different solutions to the same problem, stimulating critical thinking, one of the competences to develop. Further options about the final project should be analyzed.

The answers to the first question about the usefulness of the subject were all very positive, since all the students posited that this subject was very useful. Besides, most of the students perceived that the approach of the instructors to the labor market was highly valuable. In fact, one of the students was thinking about in starting a master’s degree oriented in the same topic.

Results about the emotional appraisal can be considered as highly positive, since even though students faced mobility restrictions, at the end of the day they have been able to follow on-campus or off-campus classes on demand. Hence, once experienced the advantages and drawbacks of both formats, 2019–2020 students have had the possibility to select an option to attend on-campus or off-campus, according students’ preferences. Results displayed in Figure 6 showed higher levels of positive feelings in almost all the pairs of assessed feelings in 2020–2021 when compared with 2019–2020 results.

### 5.2. Feedback about ‘Smart Classrooms’

As already explained, all the instruments discussed in this paper deal with getting information through open-ended students’ responses. So, there are no constraints on the students’ answers, a fact that implies getting statements that reflect freely students’ opinions, without conditioning their viewpoint. Nevertheless, a potential drawback of these approaches is that in cases where the surveyed people provide no useful information about the topics of interest for the research, no data can be collected about that topic. Here both the mastery and the experience of the interviewer have been key elements to obtain results of interest, when conducting the interviews in a professional manner which avoid influencing students’ opinions. Nevertheless, to ensure collecting feedback about the SC format, two open-ended questions specifically oriented to get students’ perceptions about SC were introduced at the end of each one of the interviews.

The empirical survey about ‘First day of class’, instrumentalized by means of two open-ended questions, offered neither opinions nor questions from students on SC or COVID-19 issues. Hence, no spontaneous concerns raised from students about SC during the first day’s session. In addition, and just before the start of the classes, several online sessions were held, attended by all the students of each of the seven ICT engineering programs with the different program coordinators. The aim of these sessions was to inform about the academic year, specifically including topics about COVID-19, the different protocols to follow at the university and the use of the SC.

Regarding the 2019–2020 BLA results, no references appeared about SC because it had not been implemented yet or about the COVID-19 issues, since the pandemic did not exist at that moment and students could finish their academic year attending classes in the campus facilities. However, these results were useful to validate that the subject had been properly designed and that there were no major issues. Since subject did not experienced major changes but the SC, it was 2020–2021 students were going to express their opinion about the subject in the same line (no major issues), allowing to get information about SC minimizing possible bias. An in deep analysis of the obtained results show that while the 2019–2020 students that attended on-line classes majorly complained the about class timetable (80%), just one of the 2020–2021 students (20%) of the 2020–2021 cohort identified timetable as a negative element. This new student’s perception could arise from one of the advantages that SC offers: the possibility of attending off-campus campus under students’ request, as highlighted in other studies [23]. On the other hand, quite surprisingly, when examining the 2020–2021 BLA survey there are not explicitly references the SC format nor to COVID-19 issues. An explanation of this latter result, once analyzed the two open-ended questions explicitly referred to SC, could be that students were quite satisfied when experiencing off-campus classes though the SC format. Hence, no spontaneous issues about SC raised from the BLA interview, in line with the results obtained by means of the ‘First day of class’ activity performed during the first day’s session.

The explicit question about SC adequacy to cope with the restrictions imposed because of the COVID-19 pandemic gave valuable information about the SC format. In fact, most students preferred to attend on-campus classes, because it is more enjoyable than the online format, especially when dealing with laboratories or offering a better students’ experience. Nevertheless, one of the students that have attended most of classes in an online format believed that SC was a very option, making no differences according his perception, attending on-campus or online off-campus classes. However, several students remarked that the SC off-campus format required more efforts to keep on concentrated, while interaction was not as fluid as attending classes on-campus classrooms. Despite some inherent issues of the SC online off-campus format, students consider that this latter option was a very good option, a finding in line with [9]. When talking about specific advantages of experiencing off-campus SC classes, two students said that attending SC off-campus classes allowed remaining comfortably at home, while not spending commuting time by one student.

The third open-ended question asked directly if learning outcomes were the same when attending on-campus or off-campus classes. Some students highlighted several elements that had a negative impact on their learning process when experiencing the SC off-campus classes: An issue highly related to interaction, since that when being off-campus students felt more reluctant to ask questions; lack of concentration; being less motivated; and having less students’ engagement. A student stressed the idea that on-campus classes allow a better adaption of instructor to the students, since there were higher levels of verbal and nonverbal communication elements when compared with attending SC off-campus classes, remarking that this issue depended a lot on the nature of each one of the subjects. Another student, that was attending all classes in the SC off-campus online format, said that he had learned a lot because he had had previously available all the written materials in advance. The fact of having written materials in advance, besides the option of having available recorded classes, were considered positive elements to enhance learning according to the viewpoint of a student.

**Figure 7:**
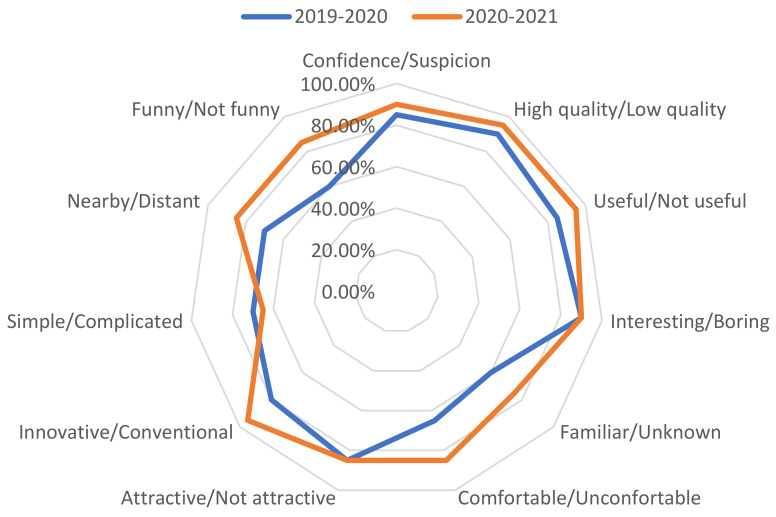
Plot of all pairs of feelings: Comparing 2019–2020 and 2020–2021 results.

Results about emotions are presented in Figure 6, while Figure 7 allows a visual comparison of both cohorts. In general, there were no great differences between both cohorts. Unexpectedly, classes are perceived as less distant and more enjoyable in 2020–2021 than in the 2019–2020 academic year. Perhaps this result could be explained by the immediately previous experience that student had in the previous semester based on an emergency remote teaching solution, clearly poorer at all levels than the SC solution. However, this latter assumption cannot explain why 2020–2021 have been superior in most of items to 2019–2020 results, when students attended classes in a F2F format. A further research should be done in this casuistic. Not surprisingly, perceptions about comfortability are higher in 2020–2021 than in 2019–2020 academic year since students had the possibility of choosing attending classes off-campus or on-campus under their own request.

### 5.3. Implications and Initial Findings of the Research

The subject was positively rated by students according to the data collected by means of the BLA interviews. Instructors, who are professionals that develop management roles in the Information Systems field, were highly valued by students. In the same line, the contents of the subject were highly appreciated, the same as the assessment logic based on Project Based Learning. One of the main issues according to the 2019–2020 students’ perceptions was the timetable, since the class sessions finish quite late. However, 2020–2021 students reflected no concerns about timetable but for one student. This result may be a direct consequence of the implementation of SC, since attending on-campus or off-campus was an option that students could decide for themselves. The open-ended question about how useful the subject was, confirmed that all students, besides being highly satisfied with the teaching and the content, believed that this subject was likely to be very useful for them in the next future.

### 5.4. Limitations of the Study and Future Research Directions

In this study, a qualitative approach was employed through getting students’ opinions by means of different techniques: A survey during the first day of class; a BLA interview, once students completed their experience on subject completion; three open-ended questions about specific topics when required, and an emotional appraisal questionnaire. All the aforementioned instruments were selected trying to avoid biases, with the aim of trying to keep students’ answers as open as possible. Besides, this research was focused on users’ perceptions as users once two students’ cohorts completed ‘Information Systems’ while the last cohort has experienced the SC format. When considering the SC format, one of the self-imposed constraints was to perform the research to students that had experienced on-campus classes at the campus facilities, which initially excluded first-year students. So, fourth-year students were the best candidates to survey, since they were the ones that had experienced on-campus classes for a longer time. When analyzing all the different available options, the subject ‘Information Systems’ was selected, a fourth-year ICT Management engineering subject that was redesigned in the 2019–2020 academic year, a redesign that was highly valued by students as shown in the 2019–2020 BLA survey. Hence, when choosing ‘Information Systems’, issues derived from the subject itself were not likely to appear in the 2020–2021 BLA survey, as in fact it happened. One of the limitations of the study was the number of enrolled students, eighteen, a potential issue that was solved by using a BLA interview without limiting the number of responses as a research instrument since this technique provides salient items from few interviews [95]. Further researches should be done to check SC perceptions in first- second- and third-year undergraduate engineering students once experienced management subjects.

## 6. Conclusions

The final goal of this work was to provide a potentially replicable model to implement redesigns of third- and fourth-year management subjects. A first stage was to implement in the design of the subject appealing and suitable methodologies to learn engineering, such as PBL, according to findings of other research works [30,31]. The second stage was to include students’ feedback to refine the design of the subject. Evidences about students’ perceptions were collected with the objective of adding additional mechanisms (see Figure 5) to allow the continuous redesign of the subject ‘Information Systems’. Additionally, information about students’ experience when using a SC was gathered to allow adjusting and improving its usage in the next academic years.

On the one hand, our results show that students of the two different cohorts are highly satisfied with the design of the subject ‘Information Systems’ according to their user experience appraisal as shown in the interviews from 2019–2020 and 2020–2021 cohorts. On the other hand, 2020–2021 students shed light about the SC format once they had experienced this format, in line with previous findings [9]. In fact, spontaneously they had no comments or complaints about following classes from home via SC. So, pre-planned specific open-ended questions asked after the BLA interview were crucial to get information about the SC format. Most of those students attended classes in the university facilities when possible, in an on-campus SC format. On-campus classes appear to have advantages over the SC off-campus format according to students’ perceptions, such as establishing more and better-quality interaction with instructors and other students, or about the class dynamics. Hence, students and instructors should put their efforts into developing new skills to minimize the mentioned limitations that the SC off-campus format has when compared with on-campus classes. However, students valued very positively different aspects that the SC introduced, such as: (1) Being able to follow off-campus classes by means of SC seems to be a very good option to cope with commuting restrictions derived from government limitations in the context of the COVID-19 pandemic or when, as a result of unexpected situation, it is impossible to be on the floor of the classroom; (2) Reviewing class sessions since it allows accessing at a specific class session on student’s convenience to watch and review some content that has not been understood by the student.

The obtained findings reflected that despite being on-campus classes the preferred option by students, the SC format in its off-campus format was highly valued as a good and versatile technological solution when facing commuting restrictions, besides offering some specific advantages, such as attending classes remotely or reviewing the specific content of a previously taught class session.

## Figures and Tables

**Table 1:** First Day of Class, ‘Information Systems’: What ‘likes’ & what ‘dislikes’ to students.

Activities	‘Likes’		‘Dislikes’	
	n	%	n	%
General overview, syllabus, content & expectations	12	92.31		
Exams, continuous assessment & grading	11	84.62		
Utility & objectives of the subject	6	46.15		
Instructor: presenting & explaining background	6	46.15		
Icebreaker: doing activities	4	30.77		
Good professor’s attitude towards students	4	30.77		
Motivating students	4	30.77		
Getting to know classmates	3	23.08		
Doing a not conventional class	2	15.38		
Advices to pass the subject	1	7.69		
Don’t exhaust the full class time	1	7.69		
Beginning subject content			10	76.92
Instructor: Poor teaching			3	23.08
Instructor: Bad attitude			3	23.08
Poor use of class time			2	15.38
Homework assignments			2	15.38

**Table 2:** ‘Information Systems’. 2019–2020 academic year. Positive common elements highlighted by the students (referenced as U1 to U5) who provided feedback.

Item	Description	Average Score	VARP ^1^	Mention Index	U1	U2	U3	U4	U5
19PCE1	Instructors work in the I. S. field	9.60	0.24	100%	10	10	9	10	9
19PCE2	An approach to labor market	9.33	0.22	60%	10	9	-	-	9
19PCE3	PBL/Study Case. Hands on	8.50	1.25	80%	8	-	9	7	10
19PCE4	Teamwork	8.00	0.00	40%	8	8	-	-	-

^1^ VARP stands for Variance for the Population.

**Table 3:** ‘Information Systems’. 2019–2020 academic year. Positive particular elements highlighted by the students who provided feedback.

Item	Description	Score	User
19PPE1	Supervising & orientating students	10	U2
19PPE2	Interaction among students & instructors	9	U2
19PPE5	Instructors: labor orientation to students	9	U3
19PPE4	Well-balanced subject: work/ECTS	9	U4
19PPE5	Proposing solutions in an Infor. System context	8.5	U2
19PPE6	Describing I.S. impact on companies	8	U2
19PPE7	Two instructors, two complementary visions	8	U3
19PPE8	A final project to assess the subject	7	U3

**Table 4:** ‘Information Systems’. 2019–2020 academic year. Negative common elements highlighted by the students (referenced as U1 to U5) who provided feedback.

Item	Description	Average Score	VARP	Mention Index	U1	U2	U3	U4	U5
19NCE1	Timetable: non-suitable	4	0.50	80%	5	4	3	-	4
19NCE2	All group deploying the same project	5	0.00	40%	5	-	-	4	5

**Table 5:** ‘Information Systems’. 2019–2020 academic year. Negative particular elements highlighted by the students who provided feedback.

Item	Description	Score	User
19NPE1	Too many ‘traditional classes’	6	U5
19NPE2	Non-dynamic classes, because of students’ attitudes	4	U3
19NPE3	Contents: too general	4	U4
19NPE4	Subject assessment: final presentation, too much weight	3	U3

**Table 6:** ‘Information Systems’. 2020–2021 academic year. Positive common elements highlighted by the students (referenced as U6 to U10) who provided feedback.

Item	Description	Average Score	VARP	Mention Index	U6	U7	U8	U9	U10
20PCE1	Instructors work in the I. S. field	9.50	0.20	100%	10	9	9	9.5	10
20PCE2	Subject: Great content	9.00	1.00	40%	-	-	10	8	-
20PCE3	PBL. Assessment System	8.67	1.56	60%	-	7	10	-	8
20PCE4	Teamwork	8.00	0.00	40%	8	-	-	-	8
20PCE5	Interactive classes	7.67	0.89	60%	7	7	-	9	-

**Table 7:** ‘Information Systems’. 2020–2021 academic year. Positive particular elements highlighted by the students who provided feedback.

Item	Description	Score	User
20PPE1	A very useful subject	10.0	U7
20PPE2	Instructors: Great teaching skills	10.0	U8
20PPE3	Two instructors, two complementary visions	8.0	U8
20PPE4	Few students per class group	8.5	U9
20PPE5	Students: developing communication skills	7.0	U10
20PPE6	Final Project: open; allowing possibilities	7.0	U10

**Table 8:** ‘Information Systems’. 2020–2021 academic year. Negative common elements highlighted by the students (referenced as U6 to U10) who provided feedback.

Item	Description	Average Score	VARP	Mention Index	U6	U7	U8	U9	U10
20NCE1	Final Project: centered in CRM	3.5	0.25	40%	4	-	-	3	-

**Table 9:** ‘Information Systems’. 2020–2021 academic year. Negative particular elements highlighted by the students who provided feedback.

Item	Description	Score	User
20NPE1	Two instructors	4	U6
20NPE2	More real cases should be presented	4	U7
20NPE3	Few assessment activities	4	U9
20NPE4	Too many ‘traditional classes’	3	U10
20NPE5	Timetable: Non-suitable timetable	1	U10

**Table 10:** List of pairs of opposite feelings assessed in the Emotional Appraisal Questionnaire.

Pairs	Emotions	Average 19–20	Average 20–21
PE1	Confidence/Suspicion	85%	90%
PE2	High Quality/Low Quality	90%	95%
PE3	Useful/Useless	85%	95%
PE4	Interesting/Boring	90%	90%
PE5	Known/Unknown	60%	75%
PE6	Comfortable/Uncomfortable	65%	85%
PE7	Attractive/Not Attractive	85%	85%
PE8	Innovative/Conventional	80%	95%
PE9	Simple/Complex	70%	65%
PE10	Nearby/Distant	70%	85%
PE11	Funny/Not Funny	60%	85%

**Table 11:** ‘Information Systems’: Summarizing and synthesizing students’ perceptions.

Element	Pos./Neg.	Items
Instructors work in the Information System field	Pos.	19PCE1; 20PCE1
Instructors: Two complementary visions	Pos.	19PPE7; 20PPE3
Instructors: Supervising & orienting students	Pos.	19PPE1
Subject: Great content & labor oriented	Pos.	19PCE2;19PPE4; 19PPE5; 19PPE6; 20PCE2; 20PPE1
Class methodology: PBL approach	Pos.	19PCE3; 19PPE8; 20PCE3; 20PPE6
Class methodology: Interactive classes	Pos.	19PPE2; 20PCE5
Competences: Teamwork	Pos.	19PCE4; 20PCE4
Competences: Soft skills	Pos.	20PPE2; 20PPE5
Few students per class	Pos.	20PPE4
Timetable: Non-suitable	Neg.	19NCE1; 20NPE5
Final Project: Same project; assessment…	Neg.	19NCE2; 20NCE1; 20NPE2; 20NPE3
Too many traditional classes	Neg.	19NPE1; 20NPE4
Non-dynamic classes	Neg.	19NPE2
Contents: Too general	Neg.	19NPE3

## Data Availability

Data available on request from the authors.
